# Supplementary material for: Data sharing in cancer research: A qualitative study exploring community members' preferences
Source: Cancer Med. 2024 Aug 9;13(15):e70084. doi: 10.1002/cam4.70084 (PMC11315867; doi:10.1002/cam4.70084)
Supplement: Supplementary file 1 — Tables S1–S2. [file CAM4-13-e70084-s001.docx]

**Supplementary Table 1.** Characteristics of the 42 community members in the 14 co-design workshops

| Workshop # (format) | Participants | Scenario preferences reported^a^ |
| --- | --- | --- |
| 1 (Hybrid) | 1: Survivor, male, major city, born overseas  2: Survivor, female, major city, born overseas  3: Carer, male, major city  4: Survivor, female, major city, born overseas | 2, 3, 3, 4 |
| 2 (Online) | 1: Carer, female, major city  2: Carer, female, major city | 3, 3 |
| 3 (Online) | 1: Survivor, male, major city  2: No personal experience with cancer, female, major city, born overseas | 4, 4 |
| 4 (Online) | 1: Carer, male, major city  2: Survivor, female, major city  3: No personal experience with cancer, female, major city, born overseas  4: No personal experience with cancer, male, inner regional | 4, 4, 4, 4 |
| 5 (In-person) | 1: No personal experience with cancer, female, major city  2: Survivor, female, major city | 3, 4 |
| 6 (Online) | 1: Survivor, male, major city,  2: Survivor/ carer, female, major city | 2, 4 |
| 7 (Hybrid) | 1: Survivor/ carer, female, inner regional, born overseas  2: Survivor, female, major city  3: Carer, female, major city | 1, 2, 2 |
| 8 (Hybrid) | 1: Survivor, female, inner regional  2: No personal experience with cancer, male, major city, born overseas | 2, 3 |
| 9 (Online) | 1: No personal experience with cancer, female, major city  2: Survivor, female, major city  3: Survivor, female, major city  4: Survivor, female, major city | 2, 2, 3, 4 |
| 10 (Online) | 1: Survivor, female, outer regional  2: Carer, female, major city  3: No personal experience with cancer, female, major city | 1, 1, 4 |
| 11 (Online) | 1: Carer, female, inner regional  2: Carer, female, major city  3: Carer, female, major city | 2, 3, 4 |
| 12 (Hybrid) | 1: Carer, female, outer regional, born overseas  2: Carer, female, remote  3: Carer, female, major city | 2, 2, 4 |
| 13 (Online) | 1: Survivor, female, major city  2: No personal experience with cancer, female, very remote  3: Survivor, male, outer regional  4: Carer, male, major city | 1, 4, 4, 4 |
| 14 (Online) | 1: Survivor, male, outer regional  2: Carer, male, major city, born overseas  3: Carer, female, outer regional  4: Carer, female, major city | 1, 1, 4, 4 |

^a^ Numbers align with the scenarios presented in Table 1. See Table 1 for a description of each scenario

**Supplementary table 2.** Completed Consolidated Criteria for Reporting Qualitative Research (COREQ) checklist.

| Topic | Item | Guide Questions/Description | Pages |
| --- | --- | --- | --- |
| Domain 1: Research team and reflexivity | | | |
| *Personal characteristics* | | | |
| Interviewer/facilitator | 1 | Which author/s conducted the interview or focus group? | 8 |
| Credentials | 2 | What were the researcher’s credentials? (e.g., PhD, MD) | 8 |
| Occupation | 3 | What was their occupation at the time of the study? | 8 |
| Gender | 4 | Was the researcher male or female? | 8 |
| Experience and training | 5 | What experience or training did the researcher have? | 8 |
| *Relationship with participants* | | | |
| Relationship established | 6 | Was a relationship established prior to study commencement? | 7-8 |
| Participant knowledge of the interviewer | 7 | What did the participants know about the researcher? (e.g., personal goals, reasons for doing the research) | 8 |
| Interviewer characteristics | 8 | What characteristics were reported about the interviewer/facilitator? (e.g., biases, assumptions, interests in the research topic) | 8 |
| Domain 2: Study design | | | |
| *Theoretical framework* | | | |
| Methodological orientation and theory | 9 | What methodological orientation was stated to underpin the study? (e.g., grounded theory, discourse analysis, ethnography, phenomenology, content analysis) | 10 |
| *Participant selection* | | | |
| Sampling | 10 | How were participants selected? (e.g., purposive, convenience, consecutive, snowball) | 8 |
| Method of approach | 11 | How were participants approached? (e.g., face-to-face, telephone, mail, email) | 7 |
| Sample size | 12 | How many participants were in the study? | 11, Fig 1 |
| Non-participation | 13 | How many people refused to participate or dropped out? What were the reasons? | Fig 1 |
| *Setting* | | | |
| Setting of data collection | 14 | Where was the data collected? (e.g., home, clinic, workplace) | 9 |
| Presence of non-participants | 15 | Was anyone else present besides the participants and researchers? | NA |
| Description of sample | 16 | What are the important characteristics of the sample? (e.g., demographic data, dates) | 11, Table 2, Supp Table 1 |
| *Data collection* | | | |
| Interview guide | 17 | Were questions, prompts, guides provided by the authors? Was it pilot tested? | 9-10, Table 1 |
| Repeat interviews | 18 | Were repeat interviews carried out? If yes, how many? | NA |
| Audio/visual recording | 19 | Did the research use audio or visual recording to collect the data? | 9 |
| Field notes | 20 | Were field notes made during and/or after the interview or focus group? | NA |
| Duration | 21 | What was the duration of the interviews or focus groups? | 9 |
| Data saturation | 22 | Was data saturation discussed? | 8 |
| Transcripts returned | 23 | Were transcripts returned to participants for comment and/or correction? | NA |
| Domain 3: Analysis and findings | | | |
| *Data analysis* | | | |
| Number of data coders | 24 | How many data coders coded the data? | 10 |
| Description of the coding tree | 25 | Did authors provide a description of the coding tree? | Table 3 |
| Derivation of themes | 26 | Were themes identified in advance or derived from the data? | 10 |
| Software | 27 | What software, if applicable, was used to manage the data? | NA |
| Participant checking | 28 | Did participants provide feedback on the findings? | NA |
| *Reporting* | | | |
| Quotations presented | 29 | Were participant quotations presented to illustrate the themes/findings? Was each quotation identified? (e.g., participant number) | 12-20 |
| Data and findings consistent | 30 | Was there consistency between the data presented and the findings? | 12-20 |
| Clarity of major themes | 31 | Were major themes clearly presented in the findings? | 12-20 |
| Clarity of minor themes | 32 | Is there a description of diverse cases or discussion of minor themes? | 12-20 |
